# Succession Planning and Leadership Development in Nursing: A Bibliometric Analysis (2000–2023)

**DOI:** 10.1155/2024/6191008

**Published:** 2024-08-09

**Authors:** Azza K. Al Hajri

**Affiliations:** Department of Learning and Performance System The Pennsylvania State University, Pennsylvania, USA

## Abstract

Succession planning is a commonly employed term in business strategy, referring to the systematic process of transferring leadership responsibilities to another employee to ensure the seamless continuity of an organization's operations. Today, this concept has acquired importance in various industries, including healthcare, with a specific focus on nursing. Several factors led nurse managers to consider preparing potential leaders for primary leadership roles due to the shortage of nurses, significantly impacting healthcare services and patient safety. Because of the importance of this topic, this present bibliometric analysis aims to analyze research studies conducted on succession planning and leadership development in nursing from 2000 to 2023 to identify research trends, key themes, and the evolution of research during this period. The Elsevier Scopus database was utilized for this analysis. This methodology identified (*n* = 326) journal studies based on the predefined keywords and timeframe. The data derived from this bibliometric analysis offers a robust foundation for conducting a systematic review, enabling a comprehensive synthesis and evaluation of the evidence in this significant field of study.

## 1. Introduction

Succession planning is a widely used term in the business strategy that aims to transfer leadership responsibilities to another employee to ensure the organization's smooth operation [1]. Today, this concept has acquired importance in various industries, including healthcare, with a specific focus on nursing. Several factors led nurse managers to consider preparing potential leaders for primary leadership roles due to the shortage of nurses, significantly impacting healthcare services and patient safety. According to the American Nursing Association [2], a nursing shortage occurs intermittently, caused by economic crises, waves of retiring nurses, and increased demand for healthcare services. This situation worsened between 2020 and 2021 with the emergence of the Coronavirus disease 2019 (COVID-19), when the world witnessed nurses leaving their jobs as a result of being in high-stress working environments, feeling professional burnout, or seeking positions with higher salaries, which had a substantial influence on global nursing practices and patient safety [3]. According to [4], healthcare organizations waste $8 million yearly due to leadership vacancies. The same study emphasizes equipping nurses with leadership competencies as crucial to reduce recruitment and orientation expenses. Hiring nurses for leadership creates a talent pipeline, enhances clinical patient outcomes, and improves healthcare quality standards [5].

## 2. Literature Review

Succession planning is critical to organization development [6]. According to Rothwell [7], succession planning is defined as “a deliberate and systematic effort by an organization to ensure leadership continuity in key positions, retain and develop intellectual and knowledge capital for the future, and encourage individual advancement” (p.29). Why is this concept significant? Rothwell [8] emphasizes that without succession planning “organizations will have difficulty maintaining leadership continuity—or identifying appropriate leaders when a change in business strategy is necessary” (p. 29). Different models and methods have been applied in the practice of succession planning in the business sector [9, 10]. However, limited literature focuses on the implementation of succession planning within nursing.

### 2.1. The Role of Nurse Manager

Nurse managers are essential figures in healthcare, supervising nursing units within hospitals. Their responsibilities, as outlined in the literature, include ensuring quality patient care, strategic decision-making, and efficient resource management [11]. Acting as a crucial link between hospital executives and frontline nurses, they facilitate communication and alignment [12]. Effective nurse managers, as indicated by research, cultivate supportive environments, advocate for staff, and endeavor to reduce turnover rates [13, 14]. Their role is indispensable for delivering high-quality care and ensuring the success of healthcare organizations [5, 15].

### 2.2. Key Factors Highlighting the Necessity of Implementing a Succession Planning

Several key factors underline the need to implement a succession planning training program for nurses to prepare them for leadership roles. These factors include an aging workforce, nursing turnover, shortages, and more responsibilities and expectations placed on nurses [5]. These factors are a global challenge and require universal best practices to face them [15]. Numerous studies have found that turnover in nursing management significantly impacts patient safety and clinical outcomes due to the disruption it causes in the continuity of leadership and care [16, 17]. A worldwide longitudinal study identifying turnover rates across various industries found that the healthcare industry has the highest management turnover rate at 9.4%, followed by the telecommunications, energy, and information technology sectors [18].

In addition, the time and resources spent on recruiting and onboarding new nursing managers may divert attention from crucial patient-focused activities [18]. Despite nurse managers' essential role, research consistently reveals that healthcare organizations lack structured succession planning training programs [5]. As a result, they bear the heavy financial burden of recruiting new nursing leadership externally rather than developing internal talent [1].

According to Smeltzer [19], succession planning should be a broad focus where individuals are prepared for future roles, not replacing positions within organizations. Smeltzer [19] also emphasizes aligning employees' and managers' objectives with the organization's overall strategic planning to increase productivity, engagement, and commitment. This notion was also supported by Rothwell [8] as succession planning is more than knowing who will take the reins if the corporate jet crashes tomorrow. It is about growing your own talent to ensure your company's future over the long term. (p. 23)

### 2.3. Strategies for Best Practice in Nurse Manager Succession Planning

Studies have identified various strategies for ensuring the best practice of nursing succession planning. According to Trepanier and Crenshaw [5], organizations should maintain a “deep pool” of potential candidates who are prepared to receive training and step into leadership positions when opportunities arise. Charan [20] asserts that no standard rule dictates internal candidates as the “best choice” and external candidates as the “second best;” the key is to select the most suitable individual for the specific position.

In contrast, Blouin and McDonagh [18] argue that external candidates have lower success rates than internally promoted employees. Organizations must regularly record employee qualities, skills, and career goals. Demand forecasting is one of the strategies that involves preparing employees for future management positions based on the organization's anticipation of the leadership skills needed to achieve its mission [21]. In addition, engaging in discussions with potential candidates about their career growth is a valuable strategy [22]. Implement a mentorship program as an effective practice for inspiring future leaders and involve new nurses in leadership roles [23]. However, some research indicates that mentorship plans can hinder succession planning because potential candidates who could be more competent may be perceived as a threat to their mentors [5]. Preparing future nurses for leadership roles can be achieved through peer-to-peer shadowing. In this method, middle-level nurses shadow their peers, encouraging shared reflection during their activities. This helps nurse middle managers develop a thoughtful approach, moving away from impulsive responses to immediate needs, avoiding ad hoc actions, and refraining from quick judgments [24].

### 2.4. Bibliometric Analysis

Despite the importance of succession planning and leadership development in nursing, there is a scarcity of a bibliometric analysis in the literature. A bibliometric analysis offers valuable insights into publication trends, key authors, and emerging research areas. Through a bibliometric analysis, researchers can identify gaps in the literature, map the intellectual structure of the field, and inform future research directions [25]. According to Ahmi [25], a bibliometric analysis is the process of analyzing metadata known as bibliographical data, such as author names, affiliations, countries, keywords, and document sources. This information is primarily stored in research database engines such as Scopus and Web of Science. It can serve as a foundation or introduction for researchers before conducting a specific study, providing insight into recent trends and publication patterns.

A bibliometric analysis is essential in shedding light on the landscape of scholarly research within a specific topic. It offers a comprehensive approach to assess the productivity and impact of research within a particular field by evaluating the volume of publications and their corresponding citations, thereby measuring the influence and significance of scholarly contributions [26]. Moreover, a bibliometric analysis assesses the performance of various metadata involved in research, including authors, institutions, countries, and funders. This aspect, known as “performance analysis,” constitutes one of the primary components of the bibliometric analysis. In addition, the methodology uncovers critical themes and topics prevalent in the literature, along with notable trends and gaps [25]. This dimension, “science mapping,” is another key component of the bibliometric analysis. Together, performance analysis and science mapping form the foundational elements of the bibliometric analysis, providing valuable insights into the scholarly landscape [27, 28].

The bibliometric analysis focuses on utilizing technology to gather data from sources such as Scopus and Web of Science. Tools such as bibliometrix in *R* and VOSviewer help organize and analyze these data quickly and accurately. This method makes handling complex information about publications, citations, keywords, and references easier, which would be complex and time-consuming to do manually. Bibliometrics also gives a clear overview of research trends, essential themes, and connections. It helps identify key insights, gaps in knowledge, and future research directions, giving a better understanding of the academic field [26].

### 2.5. Significance of the Study

There are various reasons for the significance of this study. Firstly, it represents the first recent bibliometric analysis conducted on nurse manager succession planning and leadership development, addressing a notable gap in the existing literature. Secondly, individuals interested in nursing succession planning and leadership development can utilize this study as a comprehensive reference to understand recent trends, identify essential papers, and recognize active authors in the field. Moreover, doctoral students focusing on nursing succession planning and leadership development can use the findings to identify potential supervisors and gain insights for their dissertations. In addition, researchers aiming to conduct systematic reviews on this topic can use this study as a foundational resource, laying the groundwork for further synthesis and evaluation of evidence. Furthermore, this study serves to identify gaps in the current literature, paving the way for future research endeavors and enhancing our understanding of nurse manager succession planning and leadership development. Lastly, researchers seeking international collaboration can find this study helpful for networking and establishing connections with colleagues worldwide.

### 2.6. Research Aim and Questions

This bibliometric analysis aims to analyze research on succession planning and leadership development in nursing from 2000 to 2023 to identify trends, key themes, and the evolution of research during this period. Performance and network analyses of succession planning in nursing were conducted to answer the following research questions:

1. What pattern of publications and document citations of the articles on succession planning and leadership development in nursing from 2000 to 2023?
2. What is the pattern of contribution of various countries to the publication of succession planning and leadership development in nursing?
3. What are the influential scholars and highly cited documents on the topic of succession planning and leadership development in nursing?
4. What are the major themes of knowledge that have been explored on the topic of succession planning and leadership development in nursing?
5. What are the most active journals that publish research on succession planning and leadership development in nursing?

## 3. Materials and Methods

### 3.1. Search Strategy

This study used the bibliometric analysis to evaluate the research on succession planning and leadership development in nursing. The bibliometric analysis is a quantitative examination of written publications that explores the historical development of scientific works to assess their impact [29–31]. It analyzes metadata including the “the source title, the year the article being published, publisher name, type of documents, the title of the article, author's name, affiliations and country of the authors, abstract, keywords, and references” [32]. In addition, it helps to understand how a specific field evolves and what is new [27]. The search was conducted on April 22, 2024, utilizing the Elsevier Scopus database to retrieve publications relevant to the study's topic. While significant bibliographic databases such as Web of Science and Dimensions exist, Elsevier Scopus was selected for its comprehensive coverage of the bibliometric analysis, especially within social science [33]. López-Illescas et al. [34] state that Scopus includes approximately 70% more sources than WoS. As a result, it is expected to yield more articles than Web of Science and Dimensions.

### 3.2. Inclusion and Exclusion Criteria

The inclusion criteria consist of articles meeting the following conditions: (a) publication between 2000 and 2023, (b) peer-reviewed status, and (c) focus on nurse manager succession planning and leadership development. These criteria are not overly restrictive to ensure a broader coverage of studies on this topic. Articles failing to meet these criteria were excluded. These measures were implemented to guarantee the credibility and relevance of the selected articles, aligning with the research objectives.

### 3.3. Data Extraction and Cleaning

**Figure 1:**
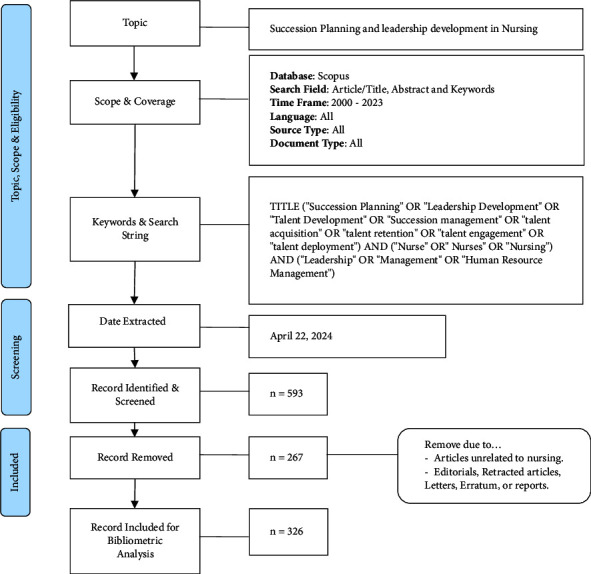
Flow diagram of the search strategy.

The author commenced the search process on the Elsevier Scopus database by exploring article titles, abstracts, and keywords to ensure a comprehensive coverage of studies on nurse manager succession planning and leadership development. The initial keyword search included terms such as “talent development,” “succession management,” “talent acquisition,” “talent retention,” “talent engagement,” and “talent deployment,” combined with “leadership,” “management,” or “human resource management,” and “nurse,” “nurses,” or “nurse,” AND “healthcare” spanning articles published from 2000 to 2023, resulting in a total of 525 documents. Further refinement of search criteria involved limiting the subject area to nursing and health professions, resulting in 593 documents in total. A thorough data cleaning review and selection process were conducted to assess the suitability of each article. This evaluation included examining content alignment with specific research objectives and ensuring the consistency and reliability of information by reviewing research titles and abstracts to confirm alignment with the research scope and adherence to inclusion criteria. A total of 267 studies were excluded for various reasons, including misalignment with the study's scope or belonging to document types such as editorials, retracted articles, letters, erratum, or reports. In addition, the search was confined to the subject areas of nursing and health professions to exclude studies from unrelated fields such as business, management, accounting, and environmental science. Ultimately, the search yielded a total of 326 documents included in this comprehensive bibliometric analysis.  Figure 1 illustrates the search process flowchart, utilizing a bibliometric analysis searching process template adapted from Zakaria et al. [32].

### 3.4. Data Analysis

Following the data cleaning phase, the metadata extracted from the 326 studies underwent an analysis utilizing five main software tools, including Microsoft Excel, OpenRefine, *R* Studio, Biblioshiny, biblioMagika, and VOSviewer. Any missing data, such as author affiliations and full names, were manually completed. Furthermore, data standardization procedures were implemented; for instance, discrepancies in author names or Scopus IDs were rectified. In addition, author affiliations were verified and added to the Excel sheet manually. Country names were also standardized; for example, variations such as “United State,” “United States,” and “USA” were unified as “United States.” Similarly, variations such as “UK” and “United Kingdom” were standardized. Once all data were cleaned and standardized, the analysis commenced using biblioMagika and Microsoft Excel, VOSviewer, and OpenRefine. The author employed OpenRefine to merge and cluster variations in authors' names, affiliations, and keywords for consistency and accuracy. Subsequently, VOSviewer was utilized to conduct network analysis, allowing for a comprehensive visualization and exploration of the relationships among the extracted data. biblioMagika was used to generate the information, tables, and overall analysis.

## 4. Results and Discussion

In this section, the results of the analyses, as described earlier, are presented in the order of the research questions. The presentation of the analyses includes graphs, tables, and the visualization of bibliometric networks using VOSviewer, accompanied by a discussion pertaining to each analysis and its corresponding results.  Table 1 displays the basic information of the documents included in the analysis. Based on the analysis from the year 2000 to 2023, the number of documents found is (*n* = 326). The total number of contributing authors in the area of succession planning and leadership development in nursing is 939. In addition, the number of cited papers is 299, whereas the total citations of the documents are 4,183.

### 4.1. Basic Information

#### 4.1.1. Document Profile

Several document types were found among the 326 documents. The majority of the documents are *articles* (*n* = 275) with a percentile of 84.36%. Second is the review documents (*n* = 34%,  = 10.43%). Then, *editorial* documents (*n* = 11%,  = 3.37%) and with *short surveys* being the least frequent (*n* = 6*%*,  = 1.84).  Table 2 displays the total publications and their percentiles.

Table 3 summarizes the languages used in nurse manager succession planning and leadership development publications. Most publications, constituting 99.08%, are in English, indicating its dominance in this field of study. Portuguese and Spanish each contribute 1.23% of the publications, while Korean and French have the lowest representation, at 0.61% and 0.31%, respectively. It is important to note that the total count is 334, as some documents are translated into multiple languages, reflecting the international dissemination of research findings and accommodating diverse linguistic audiences. This distribution highlights the prevalence of English in research publications in this area, with comparatively fewer publications available in other languages.

#### 4.1.2. Research Trends and Pattern of Citations

**Figure 2:**
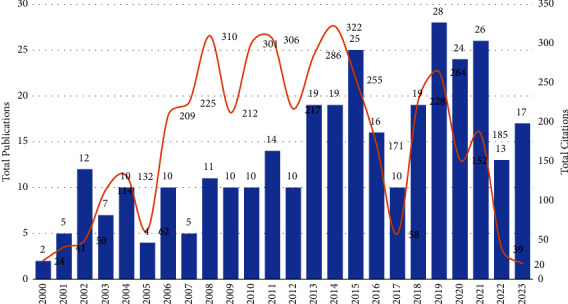
Total publication and citation per year. *Note.* Succession planning and leadership development in nursing publications per year from 2000 to 2023 (*n* = 326).

Table 4 provides insights into the evolution of research on nursing succession planning and leadership development from 2000 to 2023. A noticeable trend emerges with a consistent increase in the number of publications, indicating a growing interest in this specialized field of study. Despite fluctuations in publication numbers observed over the years, since 2008, there has been a clear upward trend. Concurrently, total citations demonstrate variability across different years since 2003, with a peak observed in 2014, reaching a substantial total of 322 citations. This peak highlights an increasing interest among authors in referencing papers about nursing succession planning and leadership development, indicative of the significance and relevance of research in this domain. Besides, various factors may have contributed to this increase in publications. One possible reason could be advancements in research methods, making it easier for researchers to conduct studies in this area. In addition, changes in healthcare policies or initiatives emphasizing the importance of leadership in nursing may have encouraged more research activities. Moreover, the growing recognition of leadership's significance in nursing and emerging challenges in the profession could have also motivated researchers' interest in exploring this topic further. These factors collectively suggest a varied landscape driving the rise in publications on nursing succession planning and leadership development.  Figure 2 graphically represents the total number of publications and citations over the years from 2000 to 2023.  Figure 2 visually displays the cumulative number of publications and citations spanning the years 2000 to 2023. The bars on the chart represent the total publications, while the line illustrates the total citations.

## 5. Country Contributions and Collaborations

**Figure 3:**
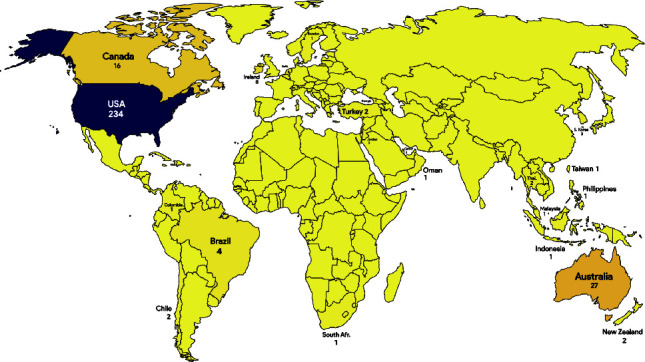
Global distribution of publications on succession planning and leadership development in nursing. *Note*. The map generated using https://iipmaps.com/map/world.

The first part of the second research question attempts to examine patterns of contributions of various countries to the publication of most countries with high publications and citations on succession planning and leadership development in nursing. According to Ahmi [25], Scopus data on countries are mainly associated with the affiliation of the authors rather than the country where the research was conducted. In fact, identifying the original country of the research typically requires reading the full article, which falls more within the scope of systematic or narrative reviews rather than the bibliometric analysis [25]. The distribution of publications in Table 5 offers an understanding into the involvement of both developed and developing countries in research on succession planning and leadership development in nursing. Developed countries such as the United States, Australia, the United Kingdom, Canada, and the Netherlands are prominent contributors to the body of the literature in this field. For instance, the United States leads with 234 publications and 2717 citations, showcasing its dominance in scholarly output and impact. Similarly, Australia, with 27 publications and 412 citations, demonstrates significant engagement in advancing knowledge and practices related to nursing succession planning and leadership development. Conversely, developing countries such as Ireland, Brazil, Hong Kong, Jordan, and South Korea also make noteworthy contributions, albeit with fewer publications compared to their developed counterparts. For example, Ireland has 8 publications and 167 citations, indicating a growing interest and engagement in addressing issues related to nursing leadership and succession planning. Despite facing challenges, these countries highlight the global nature of this research topic and the importance of diverse perspectives in advancing the field.  Figure 3 displays the global distribution map of publications on succession planning and leadership development in nursing, providing better visualization of the total publications in each country and continent.

### 5.1. Productive Authors and Cocitation Networks

To address the first part of the third research question, an authorship analysis was conducted using biblioMagika to provide valuable insights into the individuals who have made significant contributions to the field of nurse manager succession planning and leadership development in nursing.  Table 6 presents the top 10 most active scholars in this area, highlighting their respective affiliations and countries. Michael R. Bleich from Duke University and Rose O. Sherman from Florida Atlantic University emerge as the most active authors, each with nine publications. Sherman's work has greatly impacted the field, as shown by the high number of citations (265). Similarly, Maria R. Shirey, Angela Brown, and Mary Casey each have four publications, but it is noteworthy that Shirey's articles have garnered considerable attention, with a total citation count of 152. Interestingly, the list includes authors from diverse geographical locations, such as the United States, Australia, Ireland, and the United Kingdom, reflecting the global nature of research on nurse manager succession planning. This diversity in authorship highlights the collaborative and interdisciplinary efforts driving advancements in this critical area of nursing practice and education.

The second part of the third research question is about the highly cited document.  Table 7 presents the top 20 highly cited articles in nurse manager succession planning and leadership development. These articles have gained significant attention within the scholarly community, as evidenced by their high citation counts. One notable article is “Preparing Nurse Leaders for 2020” by Huston [35], which appears twice on the list and has a citation count of 182. Other influential articles include “The Essentials of Nursing Leadership: A Systematic Review of Factors and Educational Interventions Influencing Nursing Leadership” by Cummings et al. [36] with a citation count of 106 and “The Role of the Charge Nurse Manager: A Descriptive Exploratory Study” by Mccallin and Frankson [37] with a citation count of 96. These articles offer comprehensive reviews and explorations of key factors and interventions impacting nursing leadership. In addition, several articles focus specifically on leadership competencies and development within nursing practice, such as “An Integrative Review of Leadership Competencies and Attributes in Advanced Nursing Practice” by Heinen et al. [38] and “An Empowerment Framework for Nursing Leadership Development: Supporting Evidence” by Macphee et al. [39]. These articles provide knowledge of the skills and attributes required for effective leadership in nursing. It is important to mention that the “Journal of Nursing Management” and the “Journal of Nursing Administration” are the top sources for most of these articles, indicating that researchers may prioritize these journals when seeking papers on nurse manager succession planning and leadership before considering other journals. However, it is important to recognize that this conclusion is drawn from the data presented in this analysis and may vary depending on researcher interests and preferences.

### 5.2. Themes of Knowledge/Keywords Analysis

**Figure 4:**
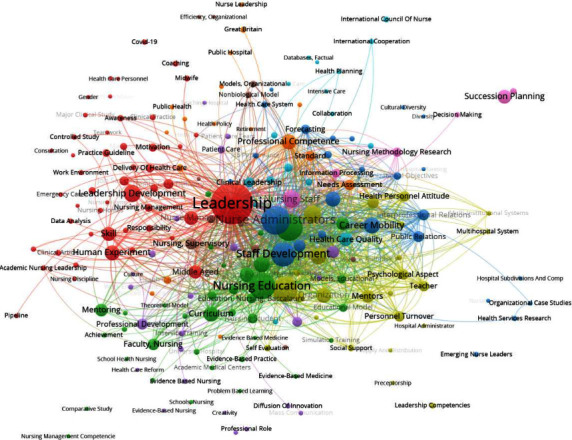
Co-occurrence network analysis of the most frequently used author keywords.

The fourth research question aims to explore into the most commonly utilized key concepts, specifically the authors' keywords, within the study of succession planning and leadership development in nursing. Identifying commonly studied concepts provide a different perspective on the conceptual structure of knowledge based within a respective boundary of data [53]. Before analyzing author keywords, all the keywords were cleaned and harmonized using OpenRefine software and manually double-checked. This step is crucial for ensuring consistency, accuracy, and standardization across the dataset, thus facilitating a more reliable and meaningful analysis of keyword trends and patterns. The process involves harmonizing synonyms, various spellings, and plurals [25]. Then, the comma-separated value file was exported to VOSviewer to start with the author keyword analysis. The type of analysis performed is co-occurrence, with the unit of analysis being *author keywords* and utilizing the counting method of *full counting*. As per the VOSviewer manual (2021), full counting assigns equal strength to each connection, while fractional counting adjusts the strength based on the number of authors involved in a document. The threshold for the minimum number of occurrences of a keyword is three out of the 1060 keywords, with 330 keywords meeting this threshold. These selected keywords, amounting to 330, are displayed in Figure 4.

As illustrated in Figure 4, the analysis of keyword co-occurrence using VOSviewer identified a total of 11 clusters based on themes. However, only the top five clusters have been selected for further discussion. The most relevant keywords within these clusters are (a) leadership in the red cluster, (b) nursing education in the green cluster, (c) staff development in the blue cluster, (d) mentoring in the gold cluster, and (e) organization innovation in the purple cluster.

First, in the red cluster, which includes keywords such as leadership, academic nursing leadership, administrative personnel, awareness, career, consultation, continuing education, curriculum, education program, future of nursing, interpersonal communication, job performance, job satisfaction, motivation, practice guidelines, team building, teamwork, thinking, training, trust, and work environment, it is notable that these keywords were included in research studies that focus on the importance of leadership and professional development initiatives in advancing the nursing profession and improving patient care outcomes. Based on these keywords, a prominent theme that emerges is Leadership and Professional Development in Nursing. It suggests a focus on enhancing leadership skills, promoting career growth, fostering effective communication and teamwork, and ensuring job satisfaction and performance among nursing professionals.

Second, the keywords grouped in the green cluster cover various important aspects of nursing. They include topics such as nursing education, clinical skills, the role of nursing teachers, improving healthcare quality, the challenges faced by middle-aged nurses, conducting nursing research, coaching methods, collaboration between healthcare workers, dealing with the effects of COVID-19, and managing finances in healthcare. The keywords in this cluster directly reflect what researchers have focused on in their studies. Based on the keywords used in this cluster, a prominent theme that emerges is the comprehensive exploration of nursing education and competency development. Researchers could explore into various aspects such as innovative teaching methods in nursing education, strategies to enhance clinical skills among nurses, initiatives to improve healthcare quality, and coping mechanisms for challenges such as the COVID-19 pandemic and resource limitations. This theme highlights the importance of continuous learning, skill-building, collaboration, and adaptation to new challenges in the nursing profession.

In the blue cluster, keywords such as staff development, professional competency, personnel selection, personnel turnover, nurse's role, nurse attitude, mentors, career, and mobility indicate a thematic focus on the development of healthcare personnel, particularly nurses. This cluster reflects an exploration of strategies and initiatives aimed at enhancing the skills, competencies, and career advancement opportunities for nursing professionals. Researchers studied various aspects, including staff training and development programs, methods to improve professional competence, approaches to personnel selection and retention, understanding the roles and attitudes of nurses in different healthcare settings, the impact of mentorship on career growth, and factors influencing career mobility within the nursing profession. This cluster highlights the importance of investing in the professional development and well-being of nurses to improve patient care outcomes and promote job satisfaction and retention in the healthcare workforce.

Then, in the gold cluster, keywords such as mentoring, nursing education research, nursing leadership, program evaluation, succession planning, capacity building, empowerment, nurse executive, outcome assessment, and strategic planning converge to indicate a thematic focus on leadership development and capacity building within the nursing profession. This cluster reflects an exploration of various topics, including the role of mentoring in supporting professional growth, research on effective nursing education strategies, leadership development initiatives, evaluation of educational programs, succession planning within nursing leadership, building organizational capacity, promoting empowerment among nursing staff, assessing outcomes of nursing interventions, and strategic planning to address future challenges in healthcare delivery. This cluster highlights the importance of nurturing leadership capabilities, fostering continuous learning and development, and strategically planning for the future to enhance the effectiveness and resilience of nursing practice and education.

Last, in the purple cluster, keywords such as organization innovation, clinical leadership, healthcare policy, in-service training, work engagement, and workload signify a thematic focus on organizational dynamics and innovation within healthcare settings. This cluster reflects an exploration of various topics, including innovative practices in healthcare organizations, effective clinical leadership strategies, the impact of healthcare policies on practice, in-service training programs for healthcare professionals, factors influencing work engagement among staff, and workload management strategies. Researchers have contributed to understand how organizational innovation, leadership practices, policy frameworks, and training programs influence work dynamics, employee engagement, and the delivery of quality care in healthcare settings. In addition, they have emphasized the importance of fostering innovation, effective leadership, and supportive work environments to enhance healthcare delivery and outcomes.

### 5.3. The Most Active Journals Publish Research

To answer the fifth research question, Table 8 presents the top 10 most active source titles for publications on nurse manager succession planning and leadership development. These source titles represent journals that have contributed significantly to the dissemination of research in this field. The top source title is the “Journal of Nursing Administration,” with 41 publications and 888 total citations. This journal, published by Wolters Kluwer Health, emerges as a key platform for scholarly discourse on nurse manager succession planning and leadership development. Following closely behind is the “Journal of Nursing Management” from Hindawi, with 29 publications and 683 total citations. This journal also serves as an important source for research in this area. Other notable source titles include “Nurse Leader” from Elsevier, “Nursing Administration Quarterly” from Wolters Kluwer Health, and the “Journal of Continuing Education in Nursing” from Slack, Inc. These publications have made significant contributions to advancing knowledge and practices related to nurse manager succession planning and leadership development.

## 6. Implication

The implications of this research are significant for both the academic and healthcare communities. The increasing interest in nurse manager succession planning and leadership development, as evidenced by the increase in publications over year, highlights the importance of addressing the impending shortage of nurse managers and cultivating essential leadership competencies. Healthcare institutions are tasked with the responsibility of proactively preparing nurses for management roles and ensuring a robust pipeline of qualified candidates. Succession planning and leadership development are strategic approaches to address this challenge.

Furthermore, international collaboration among countries contributing to this body of knowledge is a promising sign of shared expertise and potential for cross-cultural learning. However, more efforts are required by researchers in the Middle East region, as there are fewer studies published in nurse manager succession planning and leadership development compared to developed countries. Recognizing leading scholars in the field of nurse manager succession planning and leadership development helps to provide a foundation for future research collaboration. This information is also helpful to refer back to the publications of the authors to build upon existing knowledge and exploring the possibility of joint collaboration on future research studies. In addition, identifying key concepts used by authors in publishing research studies on nurse manager succession planning and leadership development, such as leadership development and clinical leadership, mentoring, and organizational innovation, researchers can uncover trends, gaps, and emerging areas of focus within the field.

## 7. Limitations

The bibliometric analysis typically focuses on the published literature indexed in specific databases, for example, Scopus, which may overlook relevant studies published in nonindexed or non-English language journals. Furthermore, the analysis may be subject to biases inherent in the selection and interpretation of data, such as keyword selection criteria or citation practices. Finally, while the bibliometric analysis can reveal trends and patterns over time, it may not capture the nuanced contextual factors that influence research trends or the practical implications of the findings. Despite these limitations, the study offers valuable future research and collaboration directions.

## 8. Conclusion and Future Direction

In conclusion, this bibliometric study sheds light on the publications of nurse manager succession planning and leadership development published from 2000 to 2023. The analysis highlights the diverse contributions of various countries to the literature on nurse manager succession planning and leadership development. While Western countries, notably the United States, have played a significant role, the emergence of contributions from smaller regions is encouraging. This contribution highlights the potential for collaborative research efforts to deepen our understanding and assess the healthcare system for the best model in nursing succession planning.

Moving forward, this research is a valuable resource for scholars, policymakers, and practitioners, providing a solid foundation for conducting systematic reviews to further synthesize and evaluate evidence in this important field. In addition, future research directions could include exploring the effectiveness of different succession planning strategies, examining the impact of leadership development programs on patient outcomes, and investigating the role of technology in enhancing leadership capabilities in nursing. These suggestions will contribute to advancing knowledge and informing practice in nurse manager succession planning and leadership development.

## Figures and Tables

**Table 1:** Basic information of the documents included in the analysis.

Metrics	Data
Start year	2000
End year	2023
Total publications	326
Number of contributing authors	939
Number of cited papers	299
Total citations	4,183
*h*-index	34
*g*-index	48
*m*-index	1.36

**Table 2:** Document type.

Document type	TP	%
Article	275	84.36
Review	34	10.43
Editorial	11	3.37
Short survey	6	1.84

**Table 3:** Languages.

Languages	TP	%
English	323	99.08
Portuguese	4	1.23
Spanish	4	1.23
Korean	2	0.61
French	1	0.31
Total	334	

*Note.* Some documents in this dataset are translated into multiple languages, accounting for the total count of 334 publications across the languages listed in the table.

**Table 4:** Publication by year.

Year	TP	NCP	TC	*h*	*g*
2000	2	2	24	1	2
2001	5	5	41	3	5
2002	12	10	50	4	6
2003	7	6	114	5	7
2004	10	10	132	6	10
2005	4	4	62	3	4
2006	10	10	209	8	10
2007	5	5	225	5	5
2008	11	11	310	8	11
2009	10	10	212	8	10
2010	10	10	301	9	10
2011	14	13	306	9	14
2012	10	8	217	7	10
2013	19	16	286	11	16
2014	19	18	322	10	17
2015	25	23	255	11	15
2016	16	16	171	7	12
2017	10	10	58	4	7
2018	19	19	228	9	14
2019	28	27	264	8	15
2020	24	22	152	7	10
2021	26	22	185	6	13
2022	13	10	39	4	5
2023	17	12	20	2	3
Total	326				

*Notes*. TP = total number of publications; NCP = number of cited publications; TC = total citations; *h* = h-index; *g* = g-index.

**Table 5:** Distribution of the 10 top countries with higher publications.

No.	Country	TP	TC
1.	United States	234	2717
2.	Australia	27	412
3.	United Kingdom	22	145
4.	Canada	16	523
5.	Ireland	8	167
6.	Netherlands	5	109
7.	Brazil	4	16
8.	Hong Kong	3	22
9.	Jordan	3	25
10.	South Korea	3	13

*Notes*. TP = total number of publications; TC = total citations.

**Table 6:** Top 10 of the most contributed scholars on nurse manager succession planning and leadership development in nursing.

Author's name	Affiliation	Country	TP	TC
Bleich, Michael R.	Duke University	USA	9	18
Sherman, Rose O.	Florida Atlantic University	USA	9	265
Shirey, Maria R.	Shirey and Associates	USA	4	152
Brown, Angela	University of Wollongong	Australia	4	31
Casey, Mary	University College Dublin	Ireland	4	88
Dewing, Jan	Queen Margaret University	UK	4	31
Young, Patricia K.	Minnesota State University	USA	4	41
Crookes, Patrick	Medicine and Health University	Australia	4	31
Campbell, Jacquelyn	Johns Hopkins University	USA	3	23
Adams, Jeffrey M.	Arizona State University	USA	3	16

**Table 7:** Top 20 highly cited articles.

No.	Authors	Title	Source title	T/C
1	Huston [35]	Preparing Nurse Leaders for 2020	Journal of Nursing Management	182
2	Cummings et al. [36]	The Essentials of Nursing Leadership: A Systematic Review of Factors and Educational Interventions Influencing Nursing Leadership	International Journal of Nursing Studies	106
3	Mccallin and Frankson [37]	The Role of the Charge Nurse Manager: A Descriptive Exploratory Study	Journal of Nursing Management	96
4	Heinen et al. [38]	An Integrative Review of Leadership Competencies and Attributes in Advanced Nursing Practice	Journal of Advanced Nursing	91
5	Macphee et al. [39]	An Empowerment Framework for Nursing Leadership Development: Supporting Evidence	Journal of Advanced Nursing	82
6	Sherman and Pross [1]	Growing Future Nurse Leaders to Build and Sustain Healthy Work Environments at the Unit Level	Online Journal of Issues in Nursing	82
7	Titzer et al. [40]	A Nurse Manager Succession Planning Model with Associated Empirical Outcomes	Journal of Nursing Administration	73
8	Redman [41]	Leadership Succession Planning: An Evidence-Based Approach for Managing the Future	Journal of Nursing Administration	59
9	Krugman and Smith [42]	Charge Nurse Leadership Development and Evaluation	Journal of Nursing Administration	59
10	Fennimore and Wolf [43]	Nurse Manager Leadership Development: Leveraging the Evidence and System-Level Support	Journal of Nursing Administration	56
11	Duygulu and Kublay [44]	Transformational Leadership Training Programme for Charge Nurses	Journal of Advanced Nursing	54
12	Stanley and Stanley [45]	Clinical Leadership and Nursing Explored: A Literature Search	Journal of Clinical Nursing	54
13	O'Neil et al. [46]	Developing Nursing Leaders: An Overview of Trends and Programs	Journal of Nursing Administration	52
14	Miles and Scott [47]	A New Leadership Development Model for Nursing Education	Journal of Professional Nursing	52
15	Dyess et al. [48]	Growing Nurse Leaders: Their Perspectives on Nursing Leadership and Today's Practice Environment	Online Journal of Issues in Nursing	51
16	Titzer et al. [49]	Nurse Manager Succession Planning: Synthesis of the Evidence	Journal of Nursing Management	50
17	Harvath et al. [50]	Enhancing Nursing Leadership in Long-Term Care. A Review of the Literature.	Research in Gerontological Nursing	49
18	Gifford et al. [51]	Managerial Leadership for Nurses' Use of Research Evidence: An Integrative Review of the Literature	Worldviews on Evidence-Based Nursing	47
19	Curtis et al. [52]	Developing Leadership in Nursing: The Impact of Education and Training	British Journal of Nursing	45
20	Griffith [15]	Effective Succession Planning in Nursing: A Review of the Literature	Journal of Nursing Management	44

**Table 8:** Top 10 of the most active source titles.

Source title	TP	TC	Publisher
Journal of Nursing Administration	41	888	Wolters Kluwer Health
Journal of Nursing Management	29	683	Hindawi
Nurse Leader	25	138	Elsevier
Nursing Administration Quarterly	25	266	Wolters Kluwer Health
Journal of Continuing Education in Nursing	20	138	Slack, Inc.
Journal of Professional Nursing	12	180	Elsevier
Nursing Management	11	47	Wolters Kluwer Health
Nursing Outlook	10	61	Elsevier
Seminars for Nurse Managers	9	24	Elsevier
Journal of Advanced Nursing	7	278	Wiley-Blackwell
